# Emerging Role of IL-10 in Hypertrophic Scars

**DOI:** 10.3389/fmed.2020.00438

**Published:** 2020-08-27

**Authors:** Zi-Li Sun, Yi Feng, Ming-Li Zou, Bin-Hong Zhao, Si-Yu Liu, Yong Du, Shun Yu, Min-Lie Yang, Jun-Jie Wu, Zheng-Dong Yuan, Guo-Zhong Lv, Ji-Ru Zhang, Feng-Lai Yuan

**Affiliations:** ^1^Wuxi Clinical Medicine School of Integrated Chinese and Western Medicine, Nanjing University of Chinese Medicine, Wuxi, China; ^2^Department of Pharmacology, Medical School, Yangzhou University, Yangzhou, China; ^3^Department of Burns and Plastic Surgery, The Affiliated Hospital of Jiangnan University, Wuxi, China

**Keywords:** interleukin 10, hypertrophic scar, inflammation, fibrosis, wound healing

## Abstract

Hypertrophic scars (HS) arise from traumatic or surgical injuries and the subsequent abnormal wound healing, which is characterized by continuous and histologically localized inflammation. Therefore, inhibiting local inflammation is an effective method of treating HS. Recent insight into the role of interleukin-10 (IL-10), an important anti-inflammatory cytokine, in fibrosis has increased our understanding of the pathophysiology of HS and has suggested new therapeutic targets. This review summarizes the recent progress in elucidating the role of IL-10 in the formation of HS and its therapeutic potential based on current research. This knowledge will enhance our understanding of the role of IL-10 in scar formation and shed new light on the regulation and potential treatment of HS.

## Introduction

A hypertrophic scar (HS) is an inevitable fibrotic consequence that occurs following trauma, surgery, burns, and inflammation (1, 2). Hypertrophic scar presents an abnormal healing process characterized by excessive cellular proliferation and aberrant extracellular matrix (ECM) deposition (i.e., types I and III collagen) (3). Hypertrophic scar is also associated with the transformation of fibroblasts into myofibroblasts. Moreover, the formation of scars can cause substantial obstacles to tissue growth, function, movement, and aesthetics, which can cause severe psychological and physiological problems in patients with HS. The incidence of HS is between 40 and 70% following surgery and as high as 91% after burns (4, 5). Although HS has been studied extensively in recent years, no practical or specific therapeutic approaches are currently available for HS, partially because the underlying mechanism remains poorly understood (6, 7).

In a skin injury, wound healing mechanisms are typically preceded by a local robust inflammatory response, which is crucial for resisting potential infection at the site where the barrier is destroyed (8). Abnormal wound repair can cause the formation of HS upon wound healing (9). Recent studies indicate that the chronic inflammatory response may be at least partially responsible for aberrant tissue repair and development of fibrosis at sites of tissue injury (10). Moreover, chronic inflammation causes the release of a large number of inflammatory mediators, which may contribute to the stimulation (profibrosis) or inhibition (antifibrosis) of fibrosis by targeting the activation of myofibroblasts, the main effector cells of HS (1, 11, 12). It is now widely believed that transforming growth factor β (TGF-β) is a central element in promoting organ fibrosis, including the skin (13), because it has a more profound effect on wound healing, which is not limited to the regulation of inflammation. In addition, reticular fibroblasts and papillary fibroblasts are present in the dermis. Only reticular fibroblasts participate in the differentiation into myofibroblasts, which is regulated by biochemical and mechanical factors. Among these factors, TGF-β1 is the main prodifferentiation mediator. Myofibroblasts represent a specific fibroblast phenotype that express α-SMA and promote the production of ECM. Myofibroblasts also induce changes to the macrophage phenotype by producing TGF-β, thereby affecting ECM degradation. Ultimately, the survival, apoptosis, and aging of myofibroblasts can directly affect scar formation (14). Similar to TGF-β, other inflammatory cytokines, such as interleukins (ILs) IL-13, IL-4, and tumor necrosis factor α (TNF-α), can indirectly regulate fibrosis, which involves macrophages as the major inflammatory cells (15). The number of myofibroblasts that differentiate from their precursors is increased in response to the activation of such TGF-β-mediated signaling pathways (16). Moreover, several inflammatory factors have been discussed as possible pathogenic mechanisms in the development of fibrosis, for example, ILs (e.g., IL-1, IL-4, IL-6, and IL-17) (17).

In contrast to TGF-β and some inflammatory mediators, other factors (e.g., IL-10) have been shown to inhibit fibrosis, including HS (18). Indeed, recent research has revealed that IL-10 can reduce skin scarring across various stages of cellular development and differentiation by inhibiting proinflammatory cytokine secretion, ECM production, and myofibroblast transdifferentiation (5, 19). Although IL-10 has been demonstrated to play a significant role in the regulation of HS formation, most IL-10 functions in scar-forming fibroblast biology have been poorly characterized. Thus, we conducted a review of the current literature on the role of IL-10 in skin scar formation, with a focus on novel findings regarding IL-10–mediated regulation of fibroblasts and myofibroblasts. This review provides novel insight into the modulation and treatment of HS.

## Biological Functions of IL-10/IL-10R

As one of the three subgroups of the IL-10 family cytokines classified by function, IL-10 is a pleiotropic candidate gene in the pathophysiological mechanism of various immune disorders (20). The IL-10 gene is located on chromosome 1 at 1q31–32, spans ~4.7 kb, and contains four introns and five exons (21). Moreover, IL-10 is a 35-kDa polypeptide cytokine that was initially purified from activated CD4^+^ T helper 2 (T_H_2) cells and was found to play a pivotal role in suppressing proinflammatory cytokine production (22, 23).

**Figure 1:**
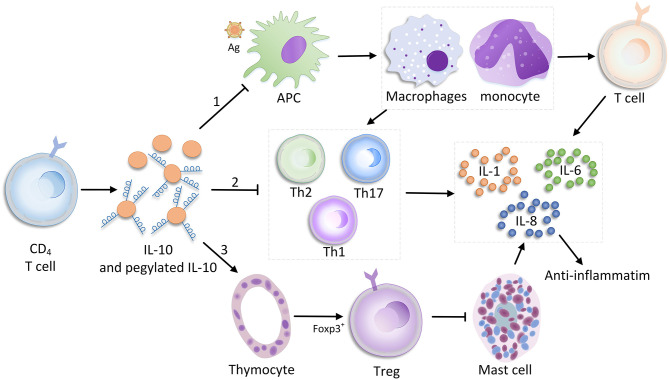
Immune-promoting and suppressing mechanisms of IL-10.

Interleukin 10 is primarily produced by CD4^+^ T cells, but also secreted by various leukocytes like macrophages, natural killer cells, B cells, dendritic cells, and neutrophils. In myeloid cells and T cells, downstream signaling is driven by various pattern recognition receptors (e.g., Toll-like receptor ligands), which regulate phosphatidylinositol-3-kinase-protein kinase B [PI (3)K-AKT], nuclear factor κB (NF-κB), tumor progression locus 2 (TPL-2)/extracellular signal–regulated kinase, and other pathways via the adaptor molecules myeloid differentiation factor 88 and TIR-domain–containing adaptor-inducing interferon β to induce IL-10 production (24, 25). In addition, IL-10 inhibits the ability of monocytes and macrophages to present antigen to T cells primarily by inhibiting the expression of histocompatibility complex class II and costimulatory molecules [e.g., CD80 (B7.1) and CD86 (B7.2)]; therefore, the expressions of other ILs (e.g., IL-1, IL-6, IL-8, and IL-12) and TNF-α are down-regulated (26–28). In addition to directly stimulating T cells, and immune-stimulating mast cells, thymocytes, and B cells, IL-10 also has an inhibitory effect on T_H_17 and T_H_2 cells due to the release of proinflammatory factors [e.g., IL-3, IL-6, and interferon γ2 (28–31)]. During bacterial infection, the antibiotic effect of IL-10 appears to be completely different. For non-MDR infections caused by highly proinflammatory bacteria, the high level of IL-10 production promotes pathogen clearance and protects the host because IL-10 regulates an excessive immune response. In contrast, IL-10 does not have a similar effect in MDR bacterial infections (32) (Figure 1).

**Figure 2:**
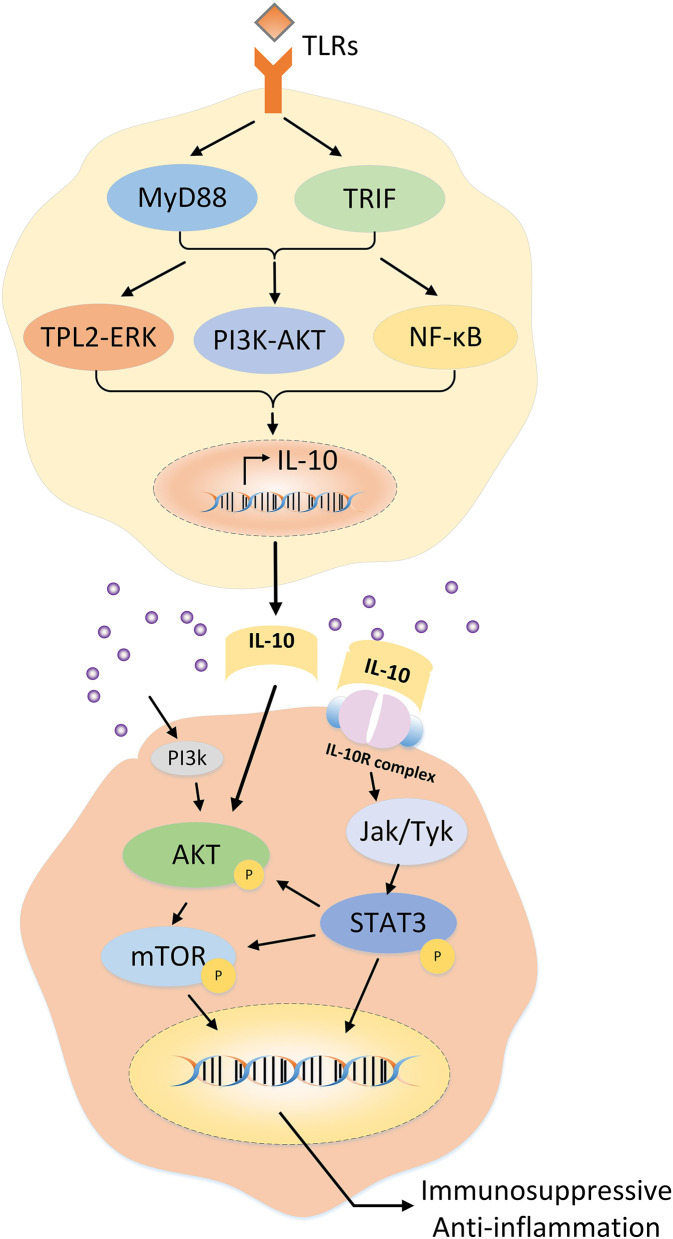
Regulation of IL-10 expression in T cells and the potential mechanism of IL-10 in hypertrophic scars.

Within the class II cytokine receptor family, as a tetramer receptor complex, the IL-10 receptor (IL-10R) consists of the IL-10α chain (IL-10Rα) and collateral IL-10β chain (IL-10Rβ) (33). Immunostaining revealed that IL-10Rα was localized and distributed on both the surface and cytoplasm of HS and HS fibroblasts (34). In addition, IL-10Rα is a high-affinity chain binding to the IL-10, whereas IL-10Rβ is involved in diverse signaling pathways related to other cytokines in the IL-10 family (e.g., IL-22, IL-26, and IL-29) (5, 35). Binding of the IL-10R complex to dimerized IL-10 results in the preferential phosphorylation of Janus kinase or tyrosine kinase, followed by activation of signal transducer and activator of transcription 3 (STAT3) and PI3K/AKT/mammalian target of rapamycin (mTOR) transcription factor pathway-mediated downstream signaling (5, 27) (Figure 2).

## IL-10 and HS

**Figure 3:**
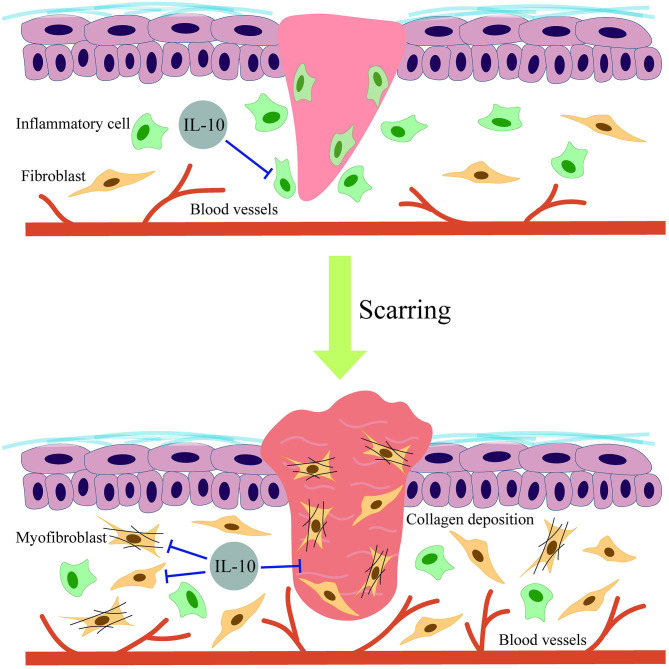
Schematic diagram of the effects of IL-10 on scarring.

Interleukin 10 is a type of anti-inflammatory cytokine that has been demonstrated to play an essential role in scar formation (36) and other fibrosis-associated diseases (17). However, the antifibrosis molecular mechanisms of IL-10 in skin scarring remain unclear. Initially, Liechty et al. (37, 38) found that IL-10–deficient mice displayed fetal wound healing with obvious inflammation and scar formation. This suggested that IL-10 may be involved by down-regulating the expression of IL-6 and IL-8. Moreover, the wounds of IL-10–deficient mice healed faster than that of normal mice (39). Another study by Gordon et al. (40) confirmed that IL-10 is highly expressed in midgestation human fetal skin in contrast to a lack of expression in post-natal human skin. The authors treated the wounds with adenoviral-mediated overexpression of IL-10 (Ad-IL-10). The results showed that the repaired Ad-IL-10–treated wounds approximated the normal dermal architecture regarding the biomechanical parameters with a reduced inflammatory response. During the same year, Peranteau et al. (41) similarly concluded that the lentivirus-mediated overexpression of IL-10 promoted wound regeneration in an adult scar formation model without abnormal collagen deposition by decreasing inflammatory mediators. More recently, a study proposed that IL-10 encoded by an orf virus contributed to skin repair in a murine full-thickness wound model (42), thereby limiting scarring. This demonstrates that purified orf virus IL-10 (ovIL-10) could promote scarless wound healing. Several studies have tried to explain the antiscarring mechanisms of IL-10. By comparing the functional differences between murine fetal and adult fibroblasts, Balaji et al. (43) demonstrated that IL-10 enhanced the migration and invasion properties of fetal fibroblasts by mediating hyaluronan synthesis. In addition, the PI3K/AKT and STAT3 signaling pathways were found to be associated with the anti-HS effects of IL-10. The results showed that IL-10 could activate AKT and STAT3 phosphorylation downstream of the IL-10, thereby accelerating the crosstalk between the PI3K/AKT and STAT3 signal transduction pathways to significantly inhibit skin fibrosis (5). Moreover, another study demonstrated that IL-10 inhibited autophagy in starved HS fibroblasts via crosstalk between the IL-10–IL-10R–STAT3 and IL-10–AKT–mTOR pathways, suggesting the therapeutic potential of IL-10 in HS (34). Recently, some studies have shown that elevated levels of IL-10 expression activated vascular endothelial growth factor (VEGF) receptor 2 (44), resulting in decreased wound inflammation and fibrosis, thereby indicating that IL-10 may mediate the antiscarring effect of VEGF receptor 2. Indeed, a study aiming to explore the antifibrosis mechanism of IL-10 in dermal fibroblasts found that IL-10 exhibited the prominent effects on increased collagen expression while decreasing the expression of matrix metalloproteinase 1 (MMP-1) and MMP-8, which inhibited the transformation of fibroblasts to myofibroblasts (18). However, the results appear to be too general and involve the use of single-cell *in vitro* experiments. In addition, there is a lack of a more wound-like model to confirm the IL-10–mediated regulation of other MMPs and ultimately whether collagen formation is affected. In conclusion, research to date has shown that the antiscarring mechanism of IL-10 may include reducing the inflammatory response; avoiding ECM overproduction (45); and regulating the migration, invasion, transformation, and apoptosis of fibroblasts (25). These diverse functions make IL-10 an indispensable component of scarless skin wound healing (Figure 3).

## IL-10–Targeting Treatment for Wound Scarring

With an increasing number of studies on the relationship between IL-10 and scarring, a growing number of therapies have been developed and applied in both human and animal models. In addition, two preclinical and phase II randomized control studies (46) proved the effectiveness of IL-10 on human wound healing. Moreover, Kieran et al. (46) created an IL-10 and IL-4 double-knockout mouse wound model, which showed skin repair with enhanced inflammation and scarring. They also studied the effect of different concentrations of recombinant human IL-10 (rhIL-10) on wound healing in both rats and humans. As a result, rhIL-10–treated rats displayed improved healing with a low inflammatory response and evident improvement of scar appearance. Similarly, human wounds treated with low concentrations of rhIL-10 exhibited the greatest recovery. Interestingly, a study on the reinnervation and revascularization of wounds treated with IL-10 observed that IL-10 increased the organization of dermal collagen, similar to that of normal skin, which suggested that IL-10 is involved in preventing HS formation (47).

Moreover, Shi et al. designed a new hybrid protein that combined human IL-10 with RGD (Arg-Gly-Asp), termed rhIL10-RGD, in an attempt to identify a more efficient antiscarring therapy. The recombinant fusion protein, IL10-RGD, which was encoded by the DNA sequence, was subcloned into a pET22b (+) vector, thereby expressing the protein in *Escherichia coli strain* BL21 (DE3). Their results showed that the antifibrotic effects of rhIL-10–RGD involved reduced ECM deposition, which may represent an effective treatment strategy for HS, with expected clinical applications (48). Furthermore, an innovative treatment was provided for scarless skin regeneration using coacervates, a tertiary complex of poly(ethylene arginyl aspartate diglyceride) (PEAD) polycation, heparin, as well as the loading of TGF-β3 and IL-10, which improved the half-lives of the growth factors, effectively increased bioactivity, and finally accelerated wound closure along with reduced scar formation (18, 49). Recent research shows that IL-10 combined with VEGF-A can promote wound closure, re-epithelialization, and collagen remodeling. However, compared with mammalian proteins, the viral proteins from the Orf virus, VEGF-E, and ovIL-10 treatment appear to cause less scarring, which was associated with greater therapeutic advantages (50). While treatment with IL-10 was primarily administered by injection, the dosage, concentration, loading approach, and combination with other drugs require additional systematic *in vivo* and *in vitro* studies. Thus, the therapeutic potential of IL-10 for HS is continuously being confirmed.

## Conclusion and Perspectives

Despite the unmet medical need, there is currently no effective method to treat or inhibit skin HS, primarily due to changes in appearance and function, impairing the quality of life of patients, both physically and psychologically. Thus, the recently available treatments for HS are insufficient, and new therapeutic approaches are needed. Because IL-10 exhibits an antiscar formation response in skin tissue, therapeutic avenues to block the IL-10/receptor in HS-associated diseases may be tailored to target this pathway. Several studies have confirmed that the application of IL-10 causes the wound edge to narrow and makes the collagen fibers in the regenerated tissue align with the collagen bundles in a more parallel orientation, without the presence of dense haphazardly arranged collagen fibers, and less red scarring. However, different methods of carrying IL-10 have mixed toxicity, while some research has confirmed that the administration of a high dose of recombinant IL-10 caused side effects, including fever, headache, malaise, and even promoted inflammation (51, 52). Because of the limitations of the wound area, most therapies are delivered by local injection, and thus the delivery of IL-10 using other methods requires further investigation. Interestingly, a method of genetic modification of rat mesenchymal stem cells to promote IL-10 delivery has been reported (53). In the future, the delivery of IL-10 through cell lines to reduce local scarring of wounds may also become a reality. Several studies have shown that M2 macrophages are also a primary target cell of IL-10 and the source of the profibrosis factor, TGF-β. M2 macrophages are associated with TGF-β-mediated fibroblast recruitment and activation and can also indirectly affect matrix proliferation and remodeling. Therefore, in addition to considering the effect of IL-10 on myofibroblasts, more attention should be paid to other cells associated with scar formation, such as reticular fibroblasts and macrophages (54, 55). Because most of the mentioned studies were performed in wound-related animal models, and not in HS models, the fibrosis-associated effects of IL-10 should be elucidated. The existing data also suggest that IL-10 may impact the antifibrotic activity in the airways through decreasing endotoxin (lipopolysaccharide)–induced inflammation and airway remodeling (56). In this review, we highlight the distinctive role of IL-10/receptor signaling in the pathophysiology of skin HS. The IL-10/receptor axis widely participates in the process of HS, demonstrating apparent direct and indirect effects on wound healing and remodeling. Generally, the IL-10/receptor signaling pathway primarily has anti-inflammatory and antiproliferative effects in the development of HS. Recently, some ILs have been approved for the clinical application in immune-related diseases. However, to date, no clinical trials have been reported on the use of IL-10 biological agents for the treatment of HS. Therefore, further studies are required to successfully translate these promising findings from *in vitro* studies and animal models into clinical practice.

## Author Contributions

F-LY, Z-LS, and YF contributed to the writing of the manuscript. M-LZ, S-YL, J-JW, B-HZ, G-ZL, YD, SY, M-LY, and Z-DY participated in the revision of the manuscript. F-LY contributed to the concept of the article. S-YL, J-JW, and G-ZL were responsible for the production of pictures and forms. M-LZ, J-RZ, and Z-LS contributed to the revision and improvement of the article. All authors contributed to the article and approved the submitted version.

## Conflict of Interest

The authors declare that the research was conducted in the absence of any commercial or financial relationships that could be construed as a potential conflict of interest.
